# A Sustainable Approach to the Metabolic Syndrome in Children and Its Economic Burden

**DOI:** 10.3390/ijerph17061891

**Published:** 2020-03-14

**Authors:** Manuel Vaquero Alvarez, Pilar Aparicio-Martinez, Francisco Javier Fonseca Pozo, Joaquín Valle Alonso, Isabel María Blancas Sánchez, Manuel Romero-Saldaña

**Affiliations:** 1 Grupo Investigación GC09 Nutrigenomics, Metabolic Syndrome, Instituto Maimónides de Investigación Biomédica de Córdoba (IMIBIC), Hospital Universitario Reina Sofía, 14071 Córdoba, Spain; h02vaalm@uco.es (M.V.A.); iblanzes_blank@hotmail.com (I.M.B.S.); 2 Grupo Investigación GC12 Clinical and Epidemiological Research in Primary Care, Instituto Maimónides de Investigación Biomédica de Córdoba (IMIBIC), Hospital Universitario Reina Sofía, 14071 Córdoba, Spain; fjfonsecapozo@yahoo.es; 3 Departamento de Enfermería, Fisioterapia y Farmacología, Universidad de Córdoba, Campus de Menéndez Pidal, 14071 Córdoba, Spain; z92rosam@uco.es; 4 Department of Emergency Medicine, Royal Bournemouth Hospital, Bournemouth BH7 7DW, UK; joa51274@hotmail.com

**Keywords:** Metabolic syndrome, pediatrics, sustainable health system

## Abstract

The prevalence of obesity continues to grow, resulting in metabolic syndrome and increasing economic burden for health systems. The objectives were to measure the ability of the NIM-MetS test, previously used in the adults, for the early and sustainable detection of the Metabolic Syndrome (MetS) in children and adolescents. Moreover, to determine the economic burden of the children with MetS. Furthermore, finally, to use and implement the NIM-MetS test, via a self-created online software, as a new method to determine the risk of MetS in children. The method used was an observational study using different instruments (NIM-MetS test, International Diabetes Federation (IDF), or Cook) and measures (body mass index). Additionally, the economic burden was estimated via a research strategy in different databases, e.g., PubMed, to identify previous papers. The results (N = 265 children, age from 10–12) showed that 23.1% had obesity and 7.2% hypertension. The prevalence of MetS using the NIM-Mets was 5.7, and the cost of these children was approximate 618,253,99 euros. Finally, a model was obtained and later implemented in a web platform via simulation. The NIM-MetS obtained is a non-invasive method for the diagnosis of risk of MetS in children.

## 1. Introduction

Nutrition has always been a central pillar of the health inside a community. Nevertheless, with the changes in agriculture and the production of food, the nutrition has also been transformed [1]. As a result, the frequency of excessive weight has increased mainly in developed countries, such as the USA, and developing countries, such as Mexico [2]. Meanwhile, the frequency of undernutrition continues, especially in developing countries, such as Cameroon [2,3,4,5]. 

According to the World Health Organization, overweight and obesity are defined as excessive fat accumulation that presents a risk to health [6]. The frequency of obesity and overweight has tripled since 1975, mainly in high-income countries, caused in high proportion by fast-food consumption and the home environment [7,8]. From that point on, the number of people suffering from overweight in the world has grown in a concerning rate, having risen to 2 billion people in 2015 [1]. The public concern is, based on that, overweight and obesity have been linked to cancer, diabetes, or heart problems [9]. Although excessive weight gain is a non-communicable disease and could be prevented, several environmental factors, such as food production, climate change, or mobility, facilitate the escalation of this issue [10,11,12]. Another part of this problem is the excessive weight gain in the younger population, mainly children and adolescents, caused by several factors, such as the shift towards motorized transportation and consumption of ultra-processed food [10,13]. 

Since 1998, the World Health Organization (WHO) has considered excessive weight gain to be a global epidemic in children, one of the most severe public health problems in the world [14]. Childhood obesity is an independent risk factor for adulthood obesity: an obese child has an 80% chance of remaining obese at 35 years old [15]. The worldwide prevalence of overweight and obese children combined has risen by 47.1% between 1980 and 2013 [16]. In Europe, the combined prevalence of overweight or obese children ranges from more than 40% in southern Europe to less than 10% in northern Europe [17]. In Spain, the ALADINO 2015 study found the prevalence of overweight children to be 23.2% and the prevalence of obese children 18.1% [18]. These results have been corroborated in Almería, southern Spain, wherein a sample representative of the population aged from 2 to 16 one third of the children and adolescents were overweight [19]. This epidemic of childhood obesity is responsible for the occurrence of metabolic diseases, previously confined to obese adults, whose body max index is equal to or over 30 [6,20]. The Metabolic Syndrome (MetS) is recognized as an escalating significant health risk in adults as well as in children and adolescents. MetS occurs in 3.3% of the pediatric population but 11.9% in overweight children and 29.2% in those obese [21]. MetS in children has been linked to the risk of cardiovascular disease during adulthood. Although, other factors such as birth weight also have been linked to MetS in children.

Moreover, a previous study carried out by Wang et al. in 2019 showed that MetS prevalence was around 2.5% in a population of Spanish adolescents [22]. Furthermore, this study and other studies showed that there are gender differences in the prevalence of MetS, being more prevalent in men than women [22,23]. Additionally, previous studies have stated that metabolic syndrome and overweight is connected with micronutrients, such as vitamin D, or social factors, such as eating with familiars or eating with the TV on [24,25]. However, to date, no unified definition exists to assess the risk of MetS in children and adolescents. Therefore, there is a lack of early diagnosis, prevention, and treatment [22].

Children with obesity and metabolic syndrome involve an economic burden. In this sense, the economic cost of the side effects of children and adolescents with overweight was rated at 14.1 billion dollars in 2010 [26]. This economic loss could be higher if the cost of climate change, especially the stress in the agriculture industry, is added to the economic burden resulted from the health effects. Furthermore, the current health programs have not included viable and sustainable to prevent or rapidly detect obesity and especially the Metabolic Syndrome (MetS) in children [1,27]. This problem is based on that there is no consensus on a MetS definition for children and adolescents. In the US, there are over 40 different definitions of MetS, and this can lead to confusion in pediatric health departments [28]. Therefore, the definition of metabolic syndrome could be selected based on the most common method of determination, which are Cook et al. [29] and de Ferranti et al. [30], International Diabetes Federation (IDF) [31], and NCEP-ATPIII [32]. The early diagnosis of MetS requires an extended timeframe and is costly, as each parameter of MetS must be investigated. A good screening test should be both highly predictive and easy to perform and interpret [31,33]. Based on all the previous statements, early diagnosis of children with a high risk of developing MetS, and type 2 diabetes mellitus or cardiovascular disease later on in life, is highly relevant. 

The authors had previously developed a new method for the early detection of MetS in the working population, which was free of biomarkers (non-invasive) and based on anthropometric variables [34,35]. This method uses non-invasive techniques and is based on just two anthropometric variables: Waist-to-Height Ratio (WtHR) (≥0.55) and Blood Pressure (BP) (≥128/85 mmHg). This method reduces the use of blood tests for those cases in which confirmation is required. It is a versatile, economical, and easily measurable method in any healthcare setting and has elevated diagnostic accuracy, with high sensitivity, specificity, and clinical concordance with the reference test (NCEP ATP III) [32]. Based on this, the current study proposed the use of NIM-MetS as a non-invasive method to determine the risk of MetS in children. This method was previously validated and used in adults, but no data regarding its usability in children is available.

The main objective of this study was to validate the NIM-MetS test, previously used in the adult population, for the early and sustainable detection of MetS in children and adolescents. Another main objective was to know the prevalence of MetS in children and adolescents through different definition guides and assess their clinical concordance [29,30,31,32]. Additionally, as a secondary objective for the diagnostic test accuracy study NIM-MetS was compared with the NCEP-ATPIII. Moreover, an added objective was to determine the economic burden of the children with MetS in the community studied. Finally, another secondary objective was to implement the NIM-MetS test as a new method to determine the risk of MetS in children via self-created online software.

## 2. Materials and Methods 

### 2.1. Design and Sample

A cross-sectional study and a diagnostic test accuracy study using a reference population of children and adolescents aged from 6-16 years of age, enrolled in schools in a town of around 3000 inhabitants in the south of Spain. Additionally, a search of the existing evidence base using databases to enable a comparison of costs resulted from the MetS was carried out (PubMed, Web of Sciences, Scopus and Cochrane), being the research strategy (“Metabolic syndrome” AND (“children” OR “pediatric” OR “children”) AND (“economic” OR “cost”) NOT (“adults” OR “teenagers”)). The selection of these databases was based on the characteristics of these instruments since Web of sciences, PubMed, Scopus, and Cochrane have been described as the most extensive and most prominent databases in the health field including protocols, papers, protocols, reviews, or meta-analysis [36,37].

The samples were selected by a random procedure and classified by age and sex. The recruitment was carried out in-person, by the authors, from April to June in 2017 at one elementary and one high school in the town. The community selected was from an urban area close to a medium city (Cordoba), although the town selected was the small-medium size, and the children were predominantly from a common area. All the schools were in homogenous zones in terms of standards of living (socioeconomic status), with a medium gross income of 23,647 euros and an average disposable income per capita of households of 19,416 00 euros. The educational centers have different educational and pedagogical features, such as a sports center, although a school health professional or specific health area was not inside the centers. Nevertheless, the health center and general practitioner were close to the schools, and the town also has an emergency health professional.

The calculation of the sample size was made based on the prevalence of SMet (3.3%). The calculation was based on the equation based on the mean of an unknown sample (*n* $=\frac{Z_{\alpha }^{2}\times S^{2}}{d^{2}}$) [38]. The Epidat 3.1 was used to carry out the sample calculation. An 80% participation rate was estimated based on the children’s population, with an expected prevalence of MetS of 3.3% [22], a confidence level of 95%, and an accuracy of 3.75%. 

A sample of 265 children was obtained, of which 144 were boys (54.3%), all of them were white. Additionally, most of the children were from primary school (70.6%) rather than from high school (78 children). The mean age of the participant s was 11.2 (2.9) years, 11.6 in boys, and 10.9 in girls, being significant the difference of age regarding gender (*p* < 0.05).

### 2.2. Procedure

The research project obtained a favorable report from the Research Ethics Committee of Córdoba (Code 260, Reference 3407). One month before the study, a meeting was held with the parents or legal representatives, children, and teachers, where they were informed of the study voluntarily and anonymity of the data. 

The inclusion criteria focused on children between 6 and 16 years who had signed informed consent from both the student and parent or legal representative. Children and adolescents that did not meet the inclusion of the previous criteria were excluded.

The students were given two appointments at the school center for physical examination, anthropometric measurements, blood pressure readings, and an electrocardiogram. All were performed at the school by a family doctor with extensive experience. The next day they had a blood test at the local office of the municipality, take the sample by the primary care nurses. The physical examination consisted of cardiac auscultation and palpation of femoral, posterior tibialis, and dorsalis pedis pulses. 

### 2.3. Measures and Instruments

The measures are taken for the study mainly focused on physical examination, observations, electrocardiogram, and blood tests. Anthropometry measurements were performed at the school by two of the authors, both of them being family doctors: 

#### 2.3.1. Weight

The weight was measured with the Omron BF511 impedance meter in Kg. The participant was conducted to stand immobile on impedance meter until the weight was stabilized and later written down.

#### 2.3.2. Height

A portable, calibrated stadiometer, model Seca® 213, was used. The participant was in the standing position with their back adjoint with the stadiometer, and their feet placed parallel and ankles together. The head must be placed according to the Frankfort Plane so that a horizontal line is drawn through the auditory canal and the lower part of the eye’s orbit, and parallel to the floor [39].

#### 2.3.3. Body Max Index

BMI = [Weight in Kilograms/(Height in Meters × Height in Meters)]. Each student was classified according to the degree of obesity using the Melo´s tables [40] into overweight (BMI + 1 SD), obese (BMI + 2 SD), and morbidly obese (BMI + 3 SD). Such tables established overweight and obesity based on modeling according to their prevalence and comparison with previous standards [40]. Moreover, the modeling of these tables was based on a statistical analysis of z-scores. 

#### 2.3.4. Waist Circumference (WC)

Two measurements were carried out for the same participant. The participant was placed in a standing position, placing the inextensible tape measure (SECA® model 203). The circumference of the waist was measured by tracing a circumference at the midpoint between the last rib and the iliac crest. 

#### 2.3.5. Waist-to-Height Ratio 

Waist-to-Height Ratio (WHtR) was calculated as waist circumference divided by height, both measured in centimeters. 

#### 2.3.6. Blood Pressure 

Blood Pressure (BP) was measured with the child seated and using the auscultatory method with a standard aneroid sphygmomanometer. 

For BP measurements, we followed the specific recommendations of the European Hypertension Society issued in 2010 [41]. The diagnostic criteria for hypertension followed the recommendations of the last Report on the Diagnosis, Evaluation, and Treatment of High Blood Pressure in Children and Adolescents [42]. Firstly, the participant was offered to use the toilet before commencing BP measurement. They were then seated in a quiet room for 5 to 10 minutes before BP measurement with their back supported and feet uncrossed. BP was measured in the right arm and at the level of the heart. Different cuff sizes were used according to the average circumference of the arm. The BP was measured at the midpoint between the scapular acromion and the elbow olecranon, with the shoulder in a neutral position and the elbow flexed at 90 °C [43]. Two measurements were made at least 5 minutes apart, and if one or both were high (≥90th percentile), two further measurements were made, and the participant was required to return on a different day for repeat measurement under the same conditions [19]. The tables proposed by the Spanish Association of Pediatrics were used for the classification of hypertensive participant s in the study [44].

#### 2.3.7. Electrocardiogram (ECG)

This was performed through standardized technology, using a Philips TC20 electrocardiograph machine. A specialist in family and community medicine interpreted the ECG at the time; however, they were later reviewed by an expert cardiologist [45]. 

#### 2.3.8. Blood Tests

Venous blood samples were obtained after 12 hours of fasting, in the supine position, and without compression. The samples were gathered in a tube, labeled, and later carried out the blood chemistry test to analyze the basic metabolic panel and a hematocrit. The parameters that were measured were: basal glucose measured in plasma; lipid profile measuring plasma after an overnight fast (total cholesterol, HDL cholesterol, LDL cholesterol, triglycerides); urea; creatinine; ions; uric acid; C-reactive protein; red blood cell count; leukocytes and platelets in plasma. 

#### 2.3.9. Urine Test

The sample required was the second micturition in the morning and was a mid-stream sample. The sample was acquittanced the same day as the electrogram. This test required the participant to discard the first 20–25 milliliters of urine they passed, collect without interruption urine in a container, and then discard the final urine produced. 

#### 2.3.10. Prevalence of MetS

The prevalence of MetS was determined according to the following children guidelines of MetS definition: NCEP-ATPIII [32], IDF [31], Cook [29], and Ferranti [30] criteria. Finally, for the diagnostic test accuracy study, NIM-MetS [35] was compared with the NCEP-ATPIII [32].

#### 2.3.11. Economic Burden

For the determination of the economic burden, the reports from the Aladino, Ideas Foundation, and DKV foundation, as well as several statistic databases, were used to determine the cost in 2010 regarding the gross domestic product (GDP) [18,46,47,48,49]. Therefore, the results obtained were used as a variable to analyze the cost in 2017 and per child.

### 2.4. Diagnostic Criteria of Metabolic Syndrome 

The definition of the MetS was based on the criteria of Cook et al. [29] and de Ferranti et al. [30], International Diabetes Federation (IDF) [31], and NCEP-ATPIII [32]. Each definition has different criteria for the diagnosis of MetS [50]. 

In the case of Cook et al. [29], the criteria were fasting glucose ≥110 mg/dL, abdominal obesity (WC ≥ 90th percentile according to age and sex), and the presence of at least a clinical feature, including triglycerides ≥110 mg/dL, HDL-C < 40 mg/dL, and blood pressure ≥90th percentile.

The criteria for de Ferranti et al. [30] were fasting glucose ≥110 mg/dL, abdominal obesity (WC ≥ 75th percentile according to age and sex), and the presence of at least a clinical feature, including triglycerides ≥100 mg/dL, HDL-C < 5 0 mg/dL, and blood pressure ≥90th percentile.

For the IDF [31], the criteria for diagnosis of MetS were fasting glucose ≥100 mg/dL, abdominal obesity (WC ≥ 90th percentile according to age and sex), and the presence of at least a clinical feature, including triglycerides ≥150 mg/dL, HDL-C < 40 mg/dL, and systolic blood pressure ≥130th mmHg and diastolic blood pressure ≥85 mmHg.

Finally, for the last definition of NCEP-ATPIII [32] for diagnosis of MetS, the criteria were fasting glucose ≥100 mg/dL, abdominal obesity (WC ≥ 90th percentile according to age and sex), and the presence of at least a clinical feature, including triglycerides ≥110 mg/dL, HDL-C < 40 mg/dL, and blood pressure ≥90th percentile.

Based on the results, in the end, to determine the model, the definition of NCEP-ATPIII [32] was selected for the prevalence of the MetS in this sample.

### 2.5. Statistical Analysis

After obtaining all the data, the SPSS program version. 22 and EPIDAT version. 4.2 were used to analyze such information. Moreover, the different European databases were used to determine the economic burden, being analyzed in an excel shift. 

The categorical variables, such as BMI, were described by their absolute and relative frequency. To compare the goodness-of-fit to an average distribution of data from continuous or discrete quantitative variables, if N > 50, the Kolmogorov–Smirnov test was used with the Lilliefors correction and the graphical representations as histograms, P-P and Q-Q graphs; whereas if N < 50, the Shapiro–Wilk test was applied. The Levene test contrasted the homoscedasticity of variances. For the comparison of two independent arithmetic means, the Student-t-test or Mann–Whitney U test was used, as indicated. Furthermore, for the comparison of three or more independent means, the ANOVA test was used, or the Kruskal–Wallis test, depending on whether it was a parametric test or not. A posthoc analysis was performed through Bonferroni and Tukey tests. The comparison of percentages was made using the chi-square test, applying Fisher’s exact test, when indicated. For contingency tables with ordinal variables, the Mantel–Haensel Chi2 test was calculated, and the d tests of Somers, Taub, and Tau-c of Kendall. Clinical concordance has been assessed using Cohen’s Kappa index. The safety and validity indexes (sensitivity, specificity, negative, and positive predictive values, positive and negative likelihood ratios, Youden index, and diagnostic validity) were determined to measure the accuracy of the diagnostic tests. Through functions obtained by discriminant analysis, the factors that best-classified children with MetS were determined. From this data based on previous studies, the economic cost was calculated using the data from children with obesity since economic and prevalence data of the MetS is not available in the Spanish Health System A posthoc analysis was performed through Bonferroni and Tukey tests. The comparison of percentages was made using the chi-square test, applying Fisher’s exact test, when indicated. For contingency tables with ordinal variables, the Mantel–Haensel Chi2 test was calculated, and the d tests of Somers, Tau-b, and Tau-c of Kendall. Clinical concordance has been assessed using Cohen’s Kappa index. The safety and validity indexes (sensitivity, specificity, negative and positive predictive values, positive and negative likelihood ratios, Youden index, and diagnostic validity) were determined to measure the accuracy of the diagnostic tests. Through functions obtained by discriminant analysis, the factors that best-classified children with MetS were determined. From this data and based on previous studies, the economic cost was calculated using the data from children with obesity since economic and prevalence data of the MetS is not available in the Spanish Health System [48,49]. 

The analysis of equality between the matrices of both groups (with and without MetS) was carried out using the MBox test. Moreover, the discriminate capacity of the predictor variables was studied with the Wilks lambda test. For each discriminant model obtained, the safety and validity indices of diagnostic tests were analyzed. The level of statistical significance was set at an alpha error of less than 5% for all statistical tests and the 95% level of confidence for the creation of confidence intervals. 

### 2.6. Implementation of the Model via a Web Simulation

From the model obtained, the software was created to an online simulation via Html5, MySQL, and JavaScript. This software allows us to determine the risk of developing MetS in children based on the parameters (blood pressure and waist-to-height). This simulation is easy to use, ubiquitous, works under any software or operative system, and represents no economic burden for the health system.

## 3. Results

### 3.1. Initial Analysis and Obesity Frequency

**Figure 1:**
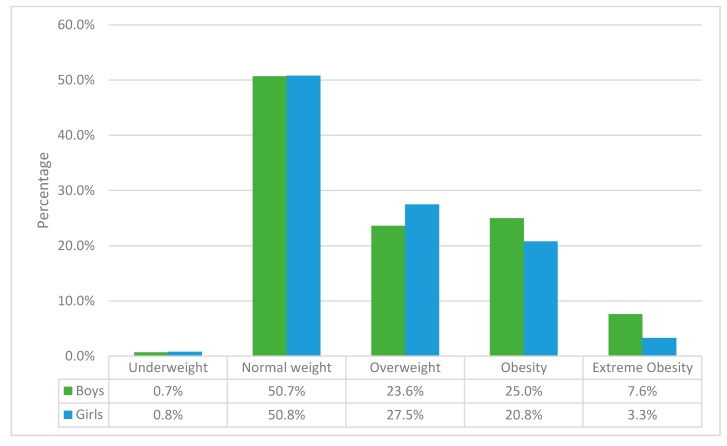
Frequency of weight according to sex.

The analysis of the data showed that obesity was 23.1% (61 children out of the 265 children). The number of children with an average weight was 134, representing 50.8 percent. According to sex, the frequencies of the weight showed similar results in girls and boys (Figure 1). However, more boys presented obesity (36 out of 144 boys) and extreme obesity (11 out of 144); meanwhile, more girls presented overweight (33 out of 121) rather than obesity, according to Melo’s tables [40] into overweight (BMI + 1 SD), obese (BMI + 2 SD) and morbidly obese (BMI + 3 SD).

**Figure 2:**
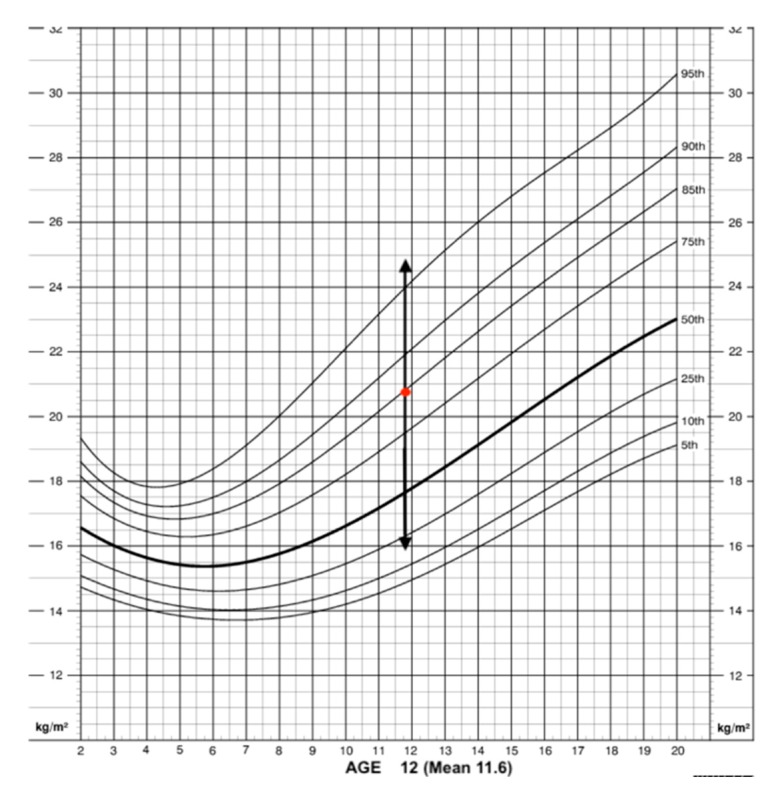
BMI according to percentiles: boys.

**Figure 3:**
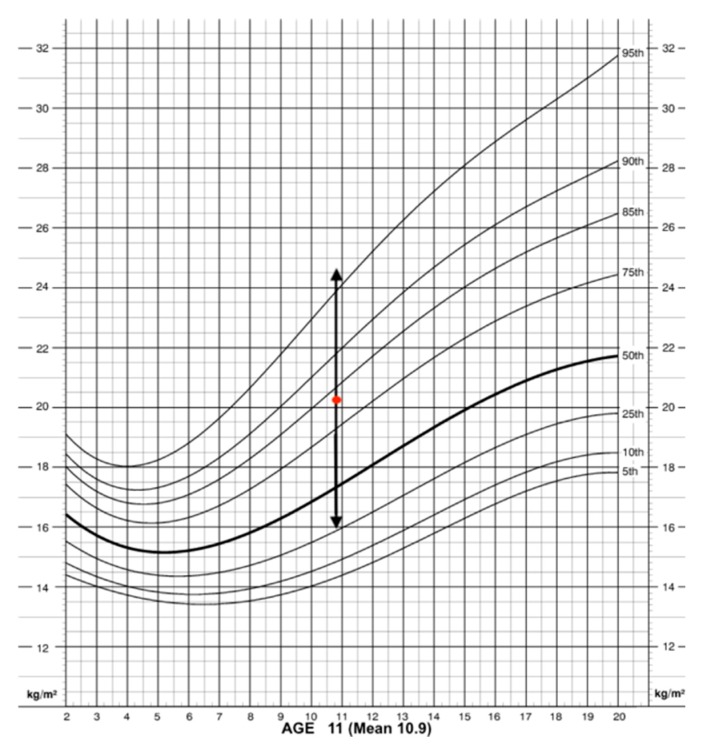
BMI according to percentiles: girls.

The BMI analyzed showed some differences between girls and boys (Figure 1). Nevertheless, the mean of the BMI according to each sex and age were average since the results were between the fifth and 85th percentile (Figure 2 and Figure 3). According to WHO, this range is associated with average weight, being over 85th equal to overweight and under fifth underweight. The mean of the BMI in the boys showed that most of them were between the 75th and 85th percentile, representing an average weight (redpoint) (Figure 2). The interquartile range in the percentiles (dark arrow) showed that a high number of boys were over the 95th percentile, meaning that they suffered from obesity.

For the girls, though the mean of BMI is lower (Table 1), the BMI is similar to the boys. These values were between the 75th and 85th percentile according to age (Figure 3). This figure showed how most girls had an average or healthy weight, represented by the red point proximate to the 85th percentile. Nevertheless, interquartile range, represented by the black arrow, showed how some girls were over the 95th percentile and therefore suffered from obesity (Figure 3). 

These results matched with no significant *p*-value (Table 1). Another variable that showed the difference between girls and boys was the waist circumference. The boys showed a higher mean of the WC than girls, and such results were supported by the *p*-value (*p* < 0.01). However, the waist-to-height Ratio showed no difference between the groups. Another variable analyzed was blood pressure, showing that most people had normal levels of blood pressure. Hypertension reached higher levels in girls than in boys (Table 1).

As for the performance of biochemical tests, blood analysis was obtained in 254 of the 265 children (95.8%). The prevalence of hypercholesterolemia was 13.2%, higher in boys (15.3%) than in girls (10.7%) without significant differences; 6.7% of the sample had low HDL cholesterol levels (<40 mg/dL) and 10.2% elevated triglycerides (≥110 mg/dL), higher in boys (13.8%) than in girls (6.8%) *p* < 0.05. 

### 3.2. Detection of Metabolic Syndrome and Economic Burden

Finally, the prevalence of MetS according to the different definition guides was: IDF (1.7%), Cook (5.1%), NCEP-ATPIII (5.1%), NIM-MetS (5.7%) and Ferranti (11%). There were no significant differences according to sex (Table 2).

The Kappa clinical concordance index between the different MetS definition guides was used. The clinical concordance between NCEP-ATPIII and Cook has been perfect (K = 1) due to the similarity of the criteria used. The highest clinical concordance was obtained between NCEP-ATPIII and Ferranti, reaching a K = 0.61 (0.43–0.78), followed by NIM-MetS with K = 0.57 (0.34–0.8) and lower with IDF K = 0.46 (0.16–0.75).

The overall clinical concordance between the MetS definition criteria, except for Cook and using the NCEP-ATPIII criteria as a reference test, was analyzed. The global Kappa index was 0.57 (0.44–0.69), finding no significant differences between the different MetS diagnostic guidelines. 

The cost derived from the children with MetS was estimated based on the economic burden of children with overweight or obesity [18,46]. The cost was 5000 million euros in 2010, and the GDP was 1,072,709 million that year, representing 0.5% of GDP from direct cost. This percentage was applied to the year 2017, in which the prevalence of obesity in children was between 22.4% and 24.6% (11,412,417 children out the 48,563,476 children in Spain) [18,46]. The expense per each child was a mean of 4,755,799 euros per year. Therefore, the cost resulted from children with MetS could be approximately 61,825,399 euros in the community studied. This cost could increase up to 72,335,718 euros based on the work if children had hypertension or other health issued problems related to the MetS [26].

### 3.3. Creation of a Model to Diagnosis the Metabolic Syndrome (MetS) in Children

A study of diagnostic tests was carried out using NCEP-ATPIII to determine the diagnostic accuracy in children of a non-invasive method of early detection of MetS (cutoff values in adults: WtHR ≥ 0.55 and BP ≥ 128/85 mmHg) (Table 3). The analysis of the NIM-MetS showed a sensitivity of 63.6% and a specificity of 97.5%, with a Youden index of 0.61 and a diagnostic validity (index of validity or proportion of participant s well diagnosed) of 95.9%. The McNemar test showed that there were no differences between the two MetS definition methods.

The NIM-MetS (adult test) obtained a diagnostic validity of 95.9%, using the NCEP-ATPIII as a reference test, but the sensitivity was 63.6%. In order to improve this disadvantage, three predictive models were obtained by discriminant analysis and were compared to choose which one could be used in a clinical decision tree, and model 1 was the preferred option. The model 1 increased the sensitivity up to 92.3% and the Youden index from 0.61 to 0.82. Therefore, it was proposed to obtain a classification model based on discriminant analysis, including the same variable variables that NIM-MetS (WtHR and BP) handled and, therefore, maintaining the essence of its non-invasive nature (without the need to use analytical variables). Three discriminant models were tested according to the result variable (grouping variable): Model 1. The grouping variable was dichotomous (MetS +/MetS -); Model 2. The grouping variable was polychotomous (three categories): 0, 1–2, and 3 or more components of MetS; Model 3. The grouping variable was ordinal polychotomous (four categories): 0, 1, 2, and 3 or more components of MetS (Table 4). The cut-off values for WHtR were 0.48 for both sexes.

**Figure 4:**
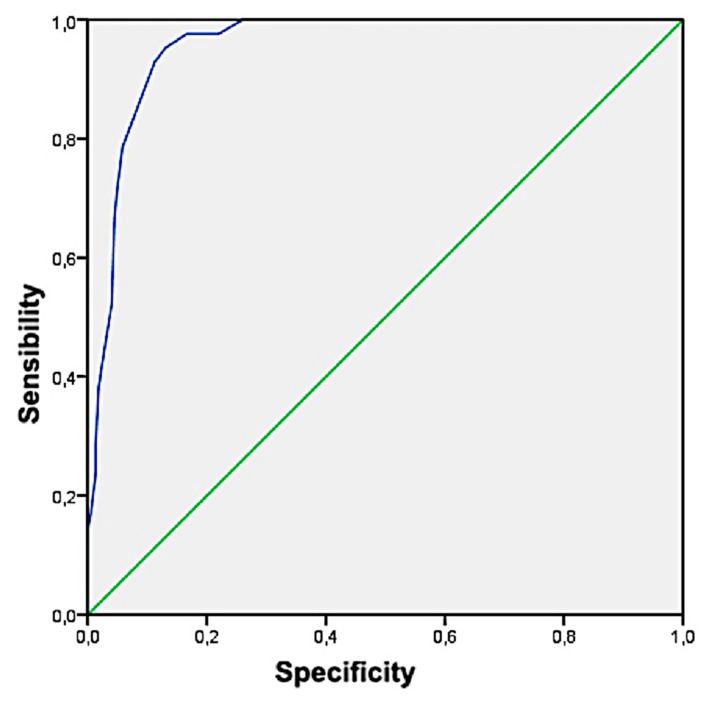
NIM-MetS Model 1 by discriminant analysis.

**Figure 5:**
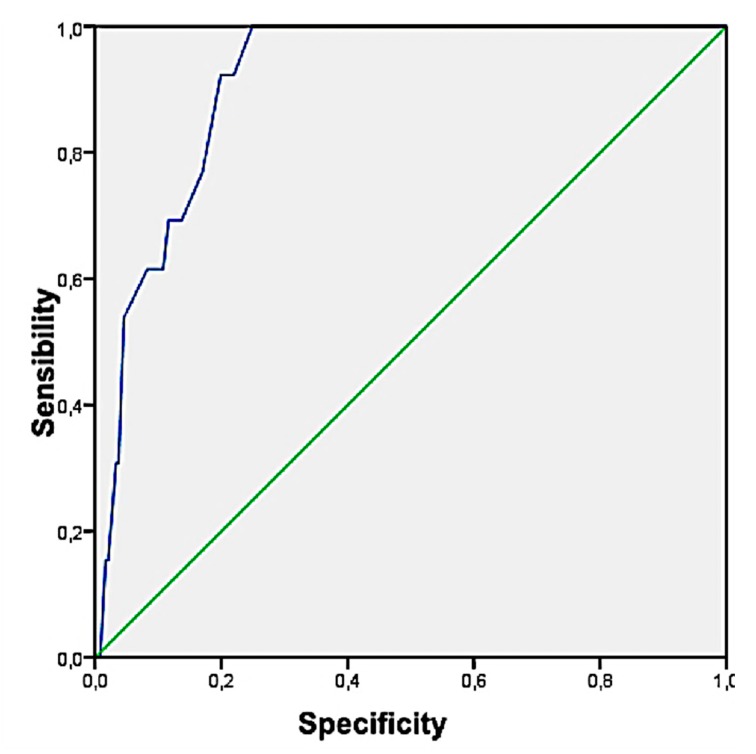
NCEP-ATPIII according to Model 1.

The results obtained showed how Model 1 obtained the highest Youden index (highest sensitivity and joint specificity) with 0.82 (0.67–0.97), a Sensitivity of 92.3% (from 74% to 100%), Specificity 89.2% (from 85.1% to 93.3%), PPV 31.6% (from 15.5% to 7.7%), NPV 99.5% (from 98.4% to 100%), LHR + 8.6 (from 5.7 to 12.7), and LHR− 0.09 (from 0.01 to 0.57). On the other hand, only Model 1 has fulfilled the theoretical assumptions (MBox and Lambda de Wilks). Finally, with the children classified as MetS according to the Model 1 of discriminant analysis, the cut-off values were obtained for the ICT variable, through the corresponding ROC curve (Figure 4 and Figure 5). The area under the curve was 95.7% (from 93.3% to 98%) and cut-off value for ICT was 0.48 (Sensitivity = 95.2% and Specificity = 87%).

Based on the previously stated, the model obtained focused on blood pressure and WC. The discriminant functions were for positive MetS (F0), and negative MetS (F1), in which DBP is the blood pressure, and the WHER is the WC which is calculated dividing the waist (cm) and height (cm) (Model 1).

F0 = A + B × WHER + C × DBP

F1 = A′ + B′ × WHER + C′ ×DBP

**Model 1.** Determination of the risk of MetS in children.

The accuracy of the model is based on the validity resulting from the sensibility, specificity, and predict values calculated. The Youden index was used to double-check the veracity of the classification and diagnosis (children with or without MetS).

### 3.4. Implementation of the Model 

After the creation of the model, to facilitate its use and, later on, validation, a web platform based on metabolic syndrome has been created. Such a platform allows the user to determine the risk of MetS based on blood pressure and WC. The platform (http://www.uco.es/4youthhealth/es/healthylife/metabolic) is available from any device and is currently available in Spanish. This platform has been created to be a free resource available to the public, being no records of who may use the platform. Moreover, the specific policies of data protection and cookies information are displayed at the bottom of the web platform.

## 4. Discussion

The current study determined the prevalence of obesity and overweight as well as MetS in children using different guidelines to compare the clinical concordance between them. The prevalence of overweight and obesity is consistent with previous studies that stated the increase of overweight and obese children from 4.2% in 1990 up to 6.7% in 2010 [51,52]. In this sense, several databases and studies have estimated that overweight and obesity are up to 24% in Spain [22,49,53,54,55]. Additionally, prior studies have noted the importance of the geographical position, and climate since the higher prevalence of overweight is more common in the Mediterranean countries [55]. Similar results were reported in the Aladin report in which the prevalence of overweight and obesity were different between the north and south of Spain. Nevertheless, less literature was found on the question of the prevalence of overweight or MetS in the pediatric age of 10 to 12 when compared to studies carried out in adults [21,22,54]. 

One interesting finding was the prevalence of MetS being 5.1%, which is in the range of 1% and 23%stablished for the MetS pediatric population. However, other studies have described that prevalence can go up to 60% amongst the obese and overweight children [28]. Based on the literature, MetS remains a controversial topic in pediatrics due to a challenging definition in pediatric populations, with more than 40 definitions for childhood MetS, most based on adaptations of adult criteria [51]. Moreover, most of the latest reviews and meta-analysis focused on determining the prevalence of adults or late teenagers using different methods. It is, therefore, not surprising that there is no consensus as to whether or how MetS should be identified in pediatric populations [3,21]. Risk factor screening and identification of pediatric populations with increased MetS risk will allow providers to detect patients at increased cardiometabolic risk and therefore play a central role in preventive pediatric care. Screening methods for metabolic syndrome in the pediatric population are of utmost importance in order to identify this group of patients and begin adequate management.

Another significant result was that the economic burden of the MetS in the pediatric age, showing an elevated that showed cost. Most literature based on adults being carried out in the USA, UK, or China, in which the MetS are increasing, and the direct and indirect cost is imperative to determine the cost-efficiency of new programs, and prevention methods [4,22]. Nonetheless, no data was found regarding the cost of the MetS in pediatric age, whether the direct or indirect cost [27]. Nevertheless, the lack of information may be limited by the strategy used since this research was not a systematic review, so, therefore, as far as the author’s knowledge, there is limited data about the economic burden. Based on that, the results from previous informs and studies were used to determine the direct cost of children with overweight. The cost based on the GDP was similar to other studies, is the cost could up to 0.6% in some areas [18,47]. A possible explanation for this difference might be the disparities between years and geographical zones. A note of caution is due here since no data currently available about the prevalence of MetS in Spain or the total cost resulted from the MetS, and the economic burden could be higher or lower.

The most relevant finding was that the validation of a method for the early detection of MetS in the pediatric population. This method proved to be useful in expanding the understanding regarding the risk of MetS, as well as how the prevalence of MetS could be determined by using anthropometric, metabolic phenotype. Such statements are based on the specificity and sensibility obtained from the model, that presents a method based purely on anthropometric, metabolic phenotype, and defined by WHtR and hypertension. Another result of the study was a difference in the prevalence of MetS according to sex, showing higher levels in males. Nevertheless, the statistical analysis was not significant, so it could consider that the prevalence was homogeneous according to sex. The distribution of participants by sex showed a minimum percentage difference in favor of males that can be considered homogeneous regarding sex. Overall, the prevalence of MetS using the NIM-Mets (5.7%) matches with a previous study that stated how the prevalence of MetS in healthy adolescent population studies in Spain was around 5% [19]. Nevertheless, the exact prevalence of MetS is difficult to identify since the prevalence was distinctive according to each definition, being from 1.6% (IDF criteria) to 5.1% (Cook et al. [29] criteria). These findings are consistent with previous studies that showed how the prevalence was from 1.7% (IDF [32] criteria) up to 11% (de Ferranti et al. [30] criteria) [50,51,56]. In this sense, this statement was also reported by González-Jiménez et al. [55] that showed how using IDF criteria in a sample of 976 Spanish children aged 10–15 years, the prevalence of MetS was 4.4% (3.85% in girls and 5.8% in boys). Moreover, the Helena Study [56] also showed the variation depending on the definition. This study showed how a cohort of 3,528 adolescents aged 12.5-17.5 years from 10 European countries reported a prevalence of MetS of 2.7% and 3.5% according to IDF and NCEP-ATPIII, respectively [56]. 

We identified the NIM-Mets as a good screening test for MetS in the pediatric population. NIM-MetS is a new method for the screening of MetS using non-invasive techniques and based on two anthropometric variables: WHtR and BP. This method reduces the use of blood tests for those cases in which confirmation is required. It has been proposed as a clinical decision tree composed of these two predictors (WHtR and BP). It is a versatile, economical, and easily measurable method in any healthcare setting with a demonstrated high diagnostic accuracy, high sensitivity, specificity, and clinic concordance with the reference test (NCEP ATP III). A simple anthropometric index to identify the status of central obesity and cardio-metabolic risk factor profiles in groups of average weight and overweight/obese children are selected based on traditional BMI criteria. Recently, an increasing number of studies have documented that WHtR and blood pressure-to-height ratio (BPHR) were suitable anthropometric indexes for the detection of obesity and hypertension in children and adolescents [33,57,58].

WHtR is superior to body mass index and waist circumference for measuring adult cardio-metabolic risk factors. A recent meta-analysis [33] demonstrated that for screening pediatric cardio-metabolic risk factors, WHtR is convenient in terms of measurement and interpretation, which is advantageous in practice and allows for the quick identification of children with cardio-metabolic risk factors at an early age. WHtR takes into account abdominal obesity and height associated with body fat accumulation or distribution. WHtR allows the same boundary value for children and adults and is easy to measure [59] and can be assessed in children and adolescents, whose height and weight change as they grow regardless of age. Finally, the last finding was the implementation of such a method to be used freely and by general practitioners, teachers, or parents. Based on these results, it could be relevant to the health field since the early implementation of prevention programs and treatment would save up to 60% of the cost of such illness [20,26,60].

These findings raise intriguing questions regarding the nature and extent of MetS in the pediatric age, the economic burden for the societies and increasing the difficulties to maintain the current health systems, the necessity of using a determined detection method the potentiality of the model created. In future investigations, it might be possible to validate the method in a higher sample, determine the prevalence of MetS in different zones in Spain as well as to determine the cost-efficiency of the model created and current economic burden in Spain for pediatric patients

## 5. Conclusions

This paper has argued how the prevalence of overweight and MetS is in the range expected for their growth rate. This study has discussed the reasons for these results may be the lack of research in pediatric age, the discordance, and the lack of a non-invasive method for the screening of MetS in children. Therefore, the current research presented the development of a new method for the screening pediatric MetS using non-invasive techniques and based on just two anthropometric variables: WHtR and BP. This method reduces the use of blood tests for those cases in which confirmation is required. It is a versatile, sustainable, and easily measurable method in any healthcare setting. This new method has shown a high diagnostic accuracy, with high sensitivity, specificity, and clinic concordance with the reference test (NCEP ATP III). Moreover, this study has identified a considerable economic burden of the MetS in children and the lack of data on the cost provoked to the national health system and GDP in Spain. These results add to the rapidly expanding field of MetS in children and the late consequences during adulthood.

This study presents some limitations that must be acknowledged. First, one source of weakness in this study, which could have affected the prevalence of MetS, was the different definitions of diagnosis and, therefore, the single criteria of each definition. Although these findings increase the knowledge in the scientific field, they are limited by the sample and geographical disposition. Based on this, a note of precaution should be addressed, since these results focused on a white population from the south of Spain. Moreover, the lack of data regarding the prevalence of MetS in children in Spain and the cost resulted from this illness also could be limited by the research strategy and this research not being a systematic review. Based on this previous state, the sources of weakness could have affected the measurements of the sustainable and cost-efficiency of the model. Concerning practical implications, this is a new non-invasive method for the screening of Pediatric MetS based on just two anthropometric variables: WtHR and BP. The new method will reduce the use of invasive tests and is an easily measurable method in any healthcare setting. The diagnostic accuracy, sensitivity, and specificity are high compared with the reference test (NCEP ATP III).

## Figures and Tables

**Table 1:** Descriptive of the anthropometric measures, blood pressure readings, and biochemical variables.

Variable	Sample (N = 265)	Boys (N = 144)	Girls (N = 121)	*p*-Value
BMI ^2^	20.5 ± 4.6	20.7 ± 4.5	20.2 ± 4.7	NS ^1^
WC	66.6 ± 11.5	68.8 ± 12	64.1 ± 10.3	<0.01
WHtR	0.454 ± 0.0061	0.46 ± 0.066	0.45 ± 0.054	0.11
BF%	2438 ± 8.6	23.6 ± 8.7	26.3 ± 8.3	<0.01
LB%	32 ± 4.4	33.5 ± 4.8	30.2 ± 3	<0.001
SBP	111.4 ± 11.3	112.8 ± 11.3	109.6 ± 11	<0.05
DBP	68.5 ± 6.3	68.1 ± 5.9	69 ± 6.7	NS ^1^
Normal BP	190 (71.7%)	103 (71.5%)	86 71%	NS ^1^
Normal-High	56 (21.1%)	32 (22.2%)	25 20.7%	NS ^1^
Hypertension	19 (7.2%)	9 (6.3%)	10 8.3%	NS ^1^
Glucose (G)	75.8 ± 6.7	76.3 ± 6.8	75.2 ± 6.6	0.64
HDL ^3^	56.8 ± 12.8	57.6 ± 13.7	56 ± 11.7	NS ^1^
HDL < 40	17 (6.7%)	8 (5.8%)	9 (7.7%)	0.29
Triglycerides	65 ± 34	63 ± 37.3	66 ± 27	NS ^1^
TG > 110	26 (10.2%)	19 (13.8%)	7 (6%)	<0.05
Cholesterol total	166.2 ± 28.8	169.1 ± 27.4	162.9 ± 30	0.09
Cholesterol ≥ 200	35 (13.2%)	22 (15.3%)	13 (10.7%)	NS ^1^
PCR	1.25 ± 4.3	1.3 ± 4.9	1.2 ± 3.4	NS ^1^

^1^ No significant *p*-value (NS); ^2^ BMI, body mass index; ^3^ HDL-Cholesterol, high-density lipoprotein-Cholesterol.

**Table 2:** Prevalence of the metabolic syndrome.

Metabolic Syndrome	Sample (N = 265)	Boys (N = 144)	Girls (N = 121)	*p*-Value
MetS [32]	13 (5.1%)	8 (5.8%)	5 (4.3%)	NS
MetS [29]	13 (5.1%)	8 (5.8%)	5 (4.3%)	NS
MetS [31]	4 (1.6%)	2 (1.6%)	2 (1.7%)	NS

**Table 3:** Diagnostic accuracy of NCEP-ATP III for MetS in childhood and adolescent population.

			Yes	No	Total
		Yes	8	6	14
PPV:	NIM-METS	No	5	235	240
		Total	13	241	254
		Indicator	Value	CI 95%	
		Sensitivity	63.6%	30.7–96.6	
		Specificity	97.5%	95.2–99.7	
		PPV ^1^	53.9%	22.9–84.8	
		NPV ^2^	98.3%	96.4–100	
		LH + ^3^	24.9	10.1–61.8	
		LH − ^3^	0.37	0.17–0.82	
		VI ^4^	95,9%	93.3–98.6	
		JI	0.61	0.3–0.9	

^1^ PPV: Positive Predictive Value, ^2^ NPV: Negative Predictive Value, ^3^ LH: Likelihood Ratio, ^4^ VI: Validity Index.

**Table 4:** Comparison of discriminant analysis models for NIM-MetS (Non-Invasive Method for early detection of Metabolic Syndrome).

Grouping Variables	Independent Variables	Variance-Covariance Matrices M Box (*p*-Value)	Wilks´s Lambda (*p*-Value)	Sensitivity (CI 95%)	Specificity (CI 95%)	Validity Index CI 95%	Youden Index CI 95%	PPV CI 95%	NPV CI 95%
**Model 1. Grouping variables (Dichotomous variable: MetS Yes, No according to NCEP criteria)**									
**MetS Yes**	WHtR	4.7 (*p* = 0.56)	0.78 (*p* < 0.001)	92.3%	86%	89.2%	0.82	31.6%	99.5%
	SBP			74%–100%	81.3%–90.6%	85.1%–93.3%	0.67–0.97	15.5%–47.7%	98.4%–100%
	DBP								
**MetS No**									
**Model 2. Grouping variables (Components of Metabolic Syndrome according to NCEP criteria)**									
**0**	WHtR								
**1–2**	SBP	73.3 (*p* < 0.001)	0.53 (*p* < 0.001)	84.6%	91.3%	90.9%	0.76	34.4%	99.1%
**≥3 (MetS yes)**	DBP		0.98 (*p* < 0.05)	61.2%–100%	84.5%–95%	87.2%–94.7%	0.56–0.96	16.4%–52.4%	97.6%–100%
**Model 3. Grouping variables (Components of Metabolic Syndrome according to NCEP criteria)**									
**0**	WHtR								
**1**	SBP	87.3 (*p* < 0.001)	0.44 (*p* < 0.001)	69.2%	95%	93.7%	0.64	42.9%	98.3%
**2**	DBP		0.97 (*p* = 0.06)	40.3%–98.2%	92.1%–98%	90.5%–96.9%	0.39–0.89	19.3%–66.4%	96.4%–100%
**≥3 (MetS yes)**			0.998 (*p* = 0.44)						
